# Translating Lung Microbiome Profiles into the Next-Generation Diagnostic Gold Standard for Pneumonia: a Clinical Investigator’s Perspective

**DOI:** 10.1128/mSystems.00153-17

**Published:** 2018-03-13

**Authors:** Georgios D. Kitsios

**Affiliations:** a Division of Pulmonary, Allergy and Critical Care Medicine, Department of Medicine, University of Pittsburgh School of Medicine and University of Pittsburgh Medical Center, Pittsburgh, Pennsylvania, USA; b Center for Medicine and the Microbiome, University of Pittsburgh, Pittsburgh, Pennsylvania, USA

**Keywords:** intensive care unit, lung microbiome, metagenomics, next-generation sequencing, pneumonia

## Abstract

Severe bacterial pneumonia is a major global cause of morbidity and mortality, yet current diagnostic approaches rely on identification of causative pathogens by cultures, which require extended incubation periods and often fail to detect relevant pathogens. Consequently, patients are prescribed broad-spectrum antibiotics in a “one-size-fits-all” manner, which may be inappropriate for their individual needs and promote antibiotic resistance. My research focuses on leveraging next-generation sequencing of microbial DNA directly from patient samples for the development of new, culture-independent definitions of pneumonia. In this perspective article, I discuss the current state of the field and focus on the conceptual and research design challenges for clinical translation. With ongoing technological advancements and application of computational biology methods for assessing clinical validity and utility, I anticipate that sequencing-based diagnostics will soon be able to positively disrupt the way we think about, diagnose, and treat pulmonary infections.

## Abstract (precis)

Severe bacterial pneumonia is a major global cause of morbidity and mortality, yet current diagnostic approaches rely on identification of causative pathogens by cultures, which require extended incubation periods and often fail to detect relevant pathogens. Consequently, patients are prescribed broad-spectrum antibiotics in a “one-size-fits-all” manner, which may be inappropriate for their individual needs and promote antibiotic resistance.

## SCOPE OF THE CLINICAL PROBLEM

In caring for patients with severe pneumonia in the intensive care unit (ICU), I am routinely faced with the frustrating challenges familiar to all clinicians treating infections: “What is the causative pathogen? Are we using the right antibiotics? Did the sputum culture results ever come back? No pathogen identified, but our patient is not getting better … Have we actually ruled out an infection? Is our patient going to recover from this?” Critical questions that frequently cannot be answered at the bedside. The root cause of the problem stems from our reliance on culture-based diagnostic tests that are neither sensitive nor fast enough to guide precise and timely treatment, resulting in empirical, suboptimal care for individual patients.

**FIG 1:**
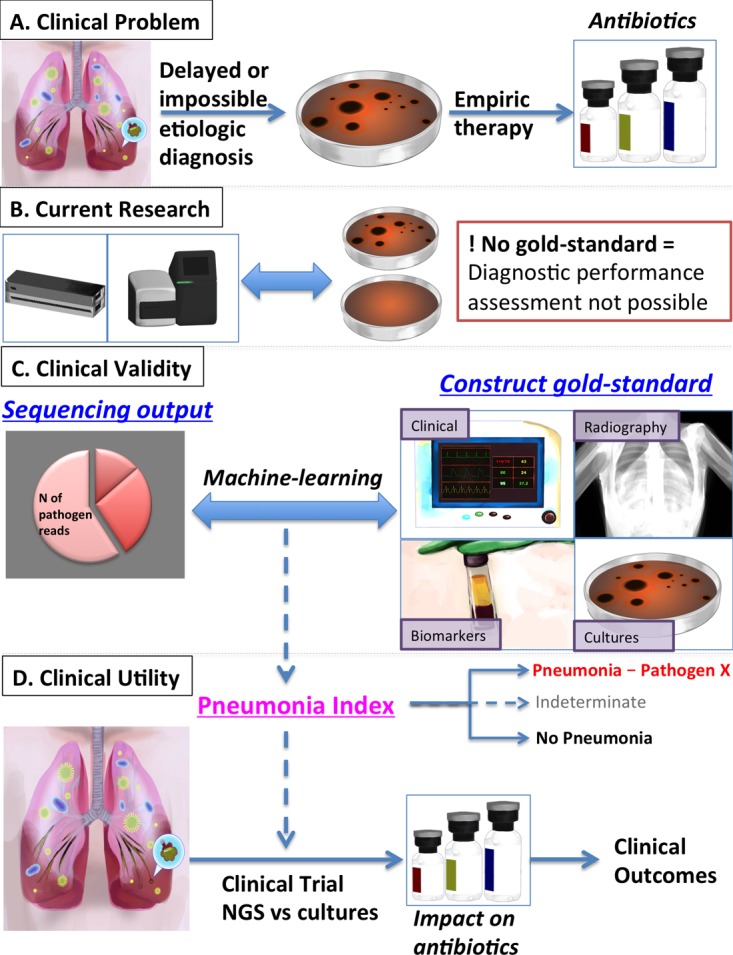
Stepwise clinical translation of next-generation sequencing (NGS) diagnostics for pneumonia. (A) Scope of the clinical problem, as delays or the inability to establish an etiologic diagnosis of bacterial pneumonia based on culture results lead to empirical one-size-fits-all antibiotic regimens. (B) Current state of research in the field with comparisons of either point-of-care or standard sequencing device outputs with clinical, culture-based diagnoses of pneumonia. The lack of a diagnostic gold standard limits our ability to assess the diagnostic performance of NGS in this context. (C) Clinical validity assessment of NGS output (and specifically metagenomic sequencing) against construction of a gold standard (incorporating clinical variables, vital signs, chest radiography scores, culture results, and validated biomarkers of injury and inflammation) with the use of machine-learning algorithms to develop a sequencing-based definition of pneumonia (pneumonia index). (D) Clinical utility assessment of the developed pneumonia index in a randomized clinical trial design of NGS (metagenomics) versus standard-of-care cultures for assessment of NGS impact on antibiotic prescriptions and clinical outcomes.

With substantial diagnostic limitations, it is not surprising that severe pneumonia has high mortality rates from 20 to 50% and long-term morbidity in ICU survivors (1). For patients presenting with fevers, sputum purulence, hypoxia, and an abnormal chest radiograph, an infectious bacterial pneumonia is rightly on top of the differential diagnosis. Along with collection of microbiologic culture specimens (respiratory secretions and/or blood samples), prompt initiation of empirical antibiotics is imperative, as even small delays translate into measurable increases in mortality (2). Following this initial encounter of clinical syndrome recognition and response, precise identification of the culprit pathogen (and its antimicrobial susceptibility) is needed to tailor further therapy. However, cultures require long incubation periods of 48 to 72 h to provide actionable results, and they frequently fail to define a causative organism (in up to 60% of cases despite systematic workup) (3), offering no specific guidance to clinicians (Fig. 1A). The resultant untailored, broad-spectrum antibiotics (typically targeting Gram-positive/negative and atypical bacteria) can be disproportionately intense, inadequate, or entirely unnecessary, depending on the causative microbial agent (4). Such intense regimens also increase the risk of toxicity, ablate indigenous protective microbiota leading to secondary infections such as *Clostridium difficile* colitis, and most concerning of all, apply selective pressure for emergence of multiresistant microbial strains, a major public health threat (5). Thus, the ability to efficiently and precisely target antibiotics is an unmet critical need in the care of severe pneumonia in the ICU, which has fueled my interest in leveraging the lung microbiome study tools for this purpose.

## RESPIRATORY MICROBIOME RESEARCH IN THE ICU

The recent wave of human lung microbiome research was made possible by the development of next-generation sequencing (NGS) techniques. Without the need for *ex vivo* growth and isolation of microbial species, amplicon-based sequencing of the highly conserved 16S rRNA gene (16S sequencing) in bacteria has uncovered bacterial communities in respiratory health and disease that are low in biomass but complex in composition, debunking long-held dogmas of “lung sterility” (6).

In proof-of-concept examinations of the validity of 16S sequencing as a diagnostic tool for pneumonia, my group (7) and others (8) have demonstrated that respiratory samples from intubated patients with pneumonia have profiles of low alpha diversity and dominance by taxa corresponding to clinically significant pathogenic organisms isolated in cultures, such as *Staphylococcus aureus* and *Klebsiella pneumoniae*. 16S sequencing has also afforded us with first-time surveys of the composition of the diagnostic “black box” that culture-negative cases represent: in our cohort, about 20% of communities are dominated by common pathogens (yet missed by cultures), and the remaining majority of cases show high abundance of bacteria with probable oral origin (e.g., *Prevotella*, *Veillonella*, etc.) (9), challenging our conventional thinking of pneumonia pathogenesis and the presumptive clinical diagnosis (7). Despite these novel insights into the ecology of pneumonia, 16S sequencing cannot be deployed quickly enough for clinical applications and does not provide information on bacterial species/strains, antibiotic resistance, or nonbacterial pathogens.

To overcome the limitations of 16S sequencing, whole metacommunity shotgun sequencing of DNA (metagenomics) or RNA (metatranscriptomics) has begun to be applied to respiratory samples to capture the wide microorganismal breadth and coding potential of pathogens with metagenomics (10, 11) and the functional activity of communities with metatranscriptomics (12, 13). With the advent of rapid, point-of-care (POC) sequencing devices (nanopore sequencing; Oxford Nanopore Technologies), bedside pathogen identification and antibiotic resistance prediction may become feasible in a matter of hours (14). In proof-of-concept case reports, investigators used nanopore sequencing competing against the clinical microbiology lab to determine who defines the causative pathogen first, and indeed they won by detecting pathogen sequences several hours before cultures provided a diagnostic signal (15). Apart from their potential for faster diagnosis, shotgun approaches offer an unprecedented opportunity for “hypothesis-free” diagnostics: with “agnostic” interrogation of metacommunity members (including viruses, fungi, or parasites), metagenomics/transcriptomics can expand our knowledge of pathogenic organisms (16), characterize poorly defined clinical syndromes (17, 18), and help identify emerging disease entities (19) across the spectrum of human pathology. However, shotgun approaches have yet to be optimized for application in respiratory samples with overwhelming amounts of human DNA compared to microbial DNA (ratio of up to 99:1) that compromise signal and analyses (11).

Shotgun approaches are not ready for clinical prime time, not just due to technical challenges but also because we still lack the diagnostic test framework to utilize their output. Feeding sequencing reports to clinicians without the obligatory (but yet to be developed) interpretive decision support would stir more confusion and clinically inappropriate decision making. As a clinical investigator, I am looking at the future research needs and the type of clinical studies that will allow us to develop actionable diagnostic tests, with a primary focus on metagenomics for the diagnosis of bacterial pneumonias that mandate targeted antibiotic therapies.

## CHALLENGES ON THE WAY TO CLINICAL TRANSLATION

Creating a clinically useful diagnostic test based on metagenomics needs to overcome several technological, practical, and cost-related challenges, including but not limited to sample preparation optimization, minimization of hands-on time, sequencing error reduction, and streamlining of analytical pipelines. Whereas such tasks are not trivial, I expect that with alignment of academic and industry interests in this field, the technical capacities of rapid sequencing devices will continue to evolve with measurable improvements in fidelity, resolution, timeliness, and cost-effectiveness within the next 5 years (20, 21). Such advancements hold the potential to change diagnostic paradigms not only for pulmonary infections but also for several infectious diseases where culture-based diagnostic approaches are currently being used (16–18). Nonetheless, from a clinical translation standpoint, the question remains: how can we move from sequencing outputs to a clinically actionable test result?

This is a formidable challenge, not unique to critical care or pulmonary infections: diagnostic performance in the absence of a gold standard, or rather, in the presence of a standard of care that is not golden (22). Metagenomic sequencing directly from patient samples can comprehensively detect viable, dead, or fastidious bacteria, whereas clinical microbiologic cultures can effectively grow only the subset of cultivable bacteria that have not been inhibited or killed by antecedent antibiotic administration. Given that metagenomics is a far more sensitive technology, conventional sensitivity/specificity analyses contrasting metagenomics with cultures are meaningless (Fig. 1B). At the same time, this “ultrasensitivity” of metagenomics can create reporting problems, as the detection of commensal communities or bacteria not typically considered pathogenic will require context-specific interpretation of their “pathogenicity” or lack thereof. To overcome these diagnostic framework challenges, we ought to apply more advanced methods for assessing clinical validity and utility (22).

## A TRANSLATIONAL ROADMAP AHEAD

### Clinical validity assessment: Can metagenomics identify the correct pathogen in pneumonia cases?

To answer the clinical validity question, the main prerequisite is knowledge of the true pathogen(s) in pneumonia, which can be unavailable in up to 60% of cases despite systematic workup (3). Thus, analyses have to be split into those with a known answer versus unknown answer.

Pathogen-confirmed cases (by cultures or rarely ancillary antigenic/antibody testing) allow for direct comparisons to derive diagnostic thresholds of sequencing output (e.g., number of specific pathogen reads, community diversity indices) associated with bacterial culture positivity above clinically accepted thresholds (e.g. >10^−4^ CFU). Culture-positive cases also offer the opportunity to refine predictive algorithms of antibiotic resistance gene detection versus clinical antibiograms (14), so that real-time antibiotic recommendations would become feasible. Consequently, observational studies of culture-confirmed cases can help develop statistical models and/or train machine-learning algorithms for sequencing-based definitions of pneumonia (pneumonia index).

In culture-negative cases, the true pathogen (if any) is unknown, and diagnosis is syndromic based on clinical constellations. To interpret the metagenomic bacterial signal in these cases, we need to refine the clinical reference standard with synthesis of multilevel data. These data can include clinical variables (e.g., vital signs, leukocytosis, sputum purulence), chest radiography scores, validated biomarkers of host inflammation (e.g., interleukin-6 and -8), alveolar epithelial injury (receptor of advanced glycation products [RAGE]), and infectious responses (procalcitonin) to be combined in “construct gold standard” pneumonia definitions. Such definitions can emerge either from supervised learning (involving expert input) of clear-cut cases (on the two ends of the pneumonia diagnosis distribution) or unsupervised classification methods identifying phenotypic classes directly from metagenomic and clinical data (22–24). With iterative training, metagenomic profiles can be translated into probabilities of pneumonia diagnosis, also incorporating prior probabilities learned from the reference culture-positive profiles. At the end of such complex algorithms, the output has to be simple and binary in order to be clinically usable: “Pneumonia by Pathogen X” or “No Pneumonia” (Fig. 1C).

### Clinical utility assessment: Does use of metagenomics in clinical practice result in better outcomes?

Demonstration of clinical efficacy for improving patient outcomes is the ultimate determinant for clinical adoption of any diagnostic test, regardless of its sophistication. The idea of “genetic exceptionalism,” i.e., the belief that genetic information is uniquely important for disease prediction over other clinically available information, did not prove to be conducive for clinical translation of genomics (25). Similarly, microbiome-based approaches have to reach standard thresholds of scientific evidence rigor to be recommended for use. Thus, metagenomics-based diagnostic tests have to be compared against standard-of-care microbiologic cultures in randomized clinical trials, anticipating that improved diagnostic accuracy with sequencing would cut down empirical and unnecessary antibiotics and result in improved (or at least noninferior) clinical response outcomes.

## CONCLUSIONS

The advent of NGS and the microbiome scientific field offer revolutionizing opportunities for entering a new, culture-independent epoch of clinical thinking, definitions, and management of infectious diseases. Ongoing technological and bioinformatic innovations, coupled with smart clinical testing and sophisticated computational biology analytics, will hopefully bring to the bedside the next-generation diagnostic tools for timely, targeted and precise antibiotic use in the ICU. Despite the challenges on the way to this culture-independent era, “the Rubicon has been crossed.”
